# Segurança medicamentosa: avanços e desafios rumo à excelência nos hospitais brasileiros

**DOI:** 10.1590/0102-311XPT120325

**Published:** 2026-04-10

**Authors:** Graça Rocha Pessoa, Lucilane Maria Sales da Silva, Jaira Gonçalves Trigueiro, Talina Carla da Silva

**Affiliations:** 1 Universidade do Estado do Rio Grande do Norte, Pau dos Ferros, Brasil.; 2 Universidade Estadual do Ceará, Fortaleza, Brasil.; 3 Instituto de Ensino do Hospital do Coração, São Paulo, Brasil.

**Keywords:** Segurança do Paciente, Cultura Organizacional, Gestão da Segurança, Pesquisa sobre Serviços de Saúde, Conduta do Tratamento Medicamentoso, Patient Safety, Organizational Culture, Safety Management, Health Services Research, Medication Therapy Management, Seguridad del Paciente, Cultura Organizacional, Administración de la Seguridad, Investigación sobre Servicios de Salud, Administración del Tratamiento Farmacológico

## Abstract

Este trabalho analisou a evolução das práticas de segurança medicamentosa no Brasil, no período de 2016 a 2023. Estudo retrospectivo documental, a partir dos relatórios da Avaliação Nacional das Práticas de Segurança do Paciente. Os dados foram coletados a partir dos gráficos de Pareto nacionais e analisados à luz da Teoria de Pareto, a qual evidencia os elementos mais vitais, responsáveis por 80% dos problemas. Constatou-se que 68% dos hospitais brasileiros com leitos de UTI aderiram à avaliação das práticas de segurança do paciente até 2023. Entre 21 indicadores, em média, 12 práticas vitais foram identificadas como fontes de não conformidades. O grau de conformidade foi maior no domínio estrutura em comparação a processo. A segurança medicamentosa esteve entre as fontes mais críticas de não conformidades. Entre 2016 e 2019, obteve melhora progressiva com redução de não conformidades de 9% para 5,82%. Contudo, a partir de 2020, tendeu à estagnação, com percentual de não conformidades mantido em cerca de 6% até 2023. A interrupção do progresso pode ser atribuída à pandemia de COVID-19, que desviou esforços e recursos para o enfrentamento da crise sanitária. Os resultados permitem compreender, com base empírica, avanços e limitações na adesão às práticas de segurança medicamentosa nos hospitais brasileiros, o que contribui para fortalecer o conhecimento sobre a maturidade atual da cultura de segurança no Brasil e subsidia a elaboração de estratégias de gestão eficazes para o SUS e para a segurança medicamentosa.

## Abstract (en)

The aim was to analyze changes in drug safety practices in Brazil from 2016 to 2023. A retrospective documental study was conducted based on reports from the Brazilian National Assessment of Patient Safety Practices. Data were collected from national Pareto graphs and analyzed in light of Pareto Theory, which states the most vital elements are responsible for 80% of problems. A total of 68% of Brazilian hospitals with ICU beds adhered to the evaluation of patient safety practices by 2023. Among 21 indicators, an average of 12 vital practices were identified as sources of noncompliance. The degree of compliance was greater in the structure domain compared to the process domain. Drug safety was among the most critical sources of noncompliance. A progressive improvement occurred between 2016 and 2019, with a reduction in noncompliance from 9% to 5.82%. Beginning in 2020, however, a tendency toward stagnation was found, with the percentage of noncompliance maintained at around 6% until 2023. The interruption in progress may be attributed to the COVID-19 pandemic, during which efforts and resources were diverted to help deal with the health crisis. The results enable an empirical understanding of advances and limitations with regards to adherence to drug safety practices at Brazilian hospitals, which contributes to strengthening knowledge on the current maturity of the safety culture in Brazil and the development of effective management strategies for the Brazilian Unified National Health System and drug safety.

## Abstract (es)

El objetivo del estudio fue analizar la evolución de las prácticas de seguridad de los medicamentos en Brasil, en el período comprendido entre 2016 y 2023. Se trata de un estudio retrospectivo documental, basado en los informes de le Evaluación Nacional de las Prácticas de Seguridad del Paciente. Los datos se recopilaron a partir de los gráficos de Pareto nacionales y se analizaron a la luz de la Teoría de Pareto, que pone de relieve los elementos más importantes, responsables del 80% de los problemas. Se constató que el 68% de los hospitales brasileños con camas de UCI se adhirieron a la evaluación de las prácticas de seguridad del paciente hasta 2023. Entre 21 indicadores, se identificaron, en promedio, 12 prácticas vitales como fuentes de incumplimientos. El grado de conformidad fue mayor en el ámbito estructural en comparación con el procesal. La seguridad de los medicamentos se encontraba entre las fuentes más críticas de incumplimientos. Entre 2016 y 2019, se observó una mejora progresiva, con una reducción de los incumplimientos del 9% al 5,82%. Sin embargo, a partir de 2020, hubo una tendencia al estancamiento, con un porcentaje de incumplimientos que se mantuvo en torno al 6% hasta 2023. La interrupción del progreso puede atribuirse a la pandemia de COVID-19, que desvió esfuerzos y recursos para hacer frente a la crisis sanitaria. Los resultados permiten comprender, con base empírica, los avances y las limitaciones en la adhesión a las prácticas de seguridad de los medicamentos en los hospitales brasileños, lo que contribuye a fortalecer el conocimiento sobre la madurez actual de la cultura de seguridad en Brasil y subsidia la elaboración de estrategias de gestión eficaces para el Sistema Único de Salud y para la seguridad de los medicamentos.

## Introdução

Todos os anos acontecem 134 milhões de eventos adversos (EAs) e mais de 2,6 milhões de pessoas morrem devido a cuidados inseguros em hospitais de países de baixa e média renda ^1^. Em dois hospitais públicos brasileiros, a densidade de incidência de EAs atingiu 1,89 por 100 pacientes/dia, dos quais, 99% foram classificados como evitáveis e 29,3% dos EAs resultaram em óbitos. EAs relacionados a medicamentos representaram 5,9% das ocorrências ^2^.

A segurança medicamentosa, definida como a ausência de danos evitáveis com o uso de medicamentos ^3^, foi incluída como área chave do Plano Global para Segurança do Paciente de 2021-2030 ^1^, reconhecida como importante fonte de risco. Embora os medicamentos sejam essenciais ao prolongamento ou à qualidade de vida, simultaneamente, podem ser inseguros e necessitam de gerenciamento eficaz para prevenção de EAs.

Desde 2013, o Ministério da Saúde implementou o Programa Nacional de Segurança do Paciente (PNSP) ^4^. A partir de 2016, o PNSP incluiu a Avaliação Nacional das Práticas de Segurança do Paciente, ato normativo que visa a gestão da qualidade nos serviços de saúde, com unidades de terapia intensiva (UTI).

Por meio dessa avaliação, os Núcleos de Qualidade e Segurança do Paciente (NQSP) respondem anualmente questionário de avaliação, que classifica o grau de adesão às boas práticas de segurança do paciente, em três níveis: (1) Conformidade alta (67%-100% de conformidade do indicador composto de adesão); (2) Conformidade média (34%-66% de conformidade do indicador composto de adesão); e (3) Conformidade baixa (0%-33% de conformidade do indicador de adesão ^5^.

O indicador composto avaliado refere-se à estrutura e ao processo. No quesito estrutura, são avaliados a existência do NQSP e do Plano de Segurança do Paciente (PSP) e a instituição dos protocolos prioritários do PNSP; no processo, o grau de adesão a protocolos específicos: adesão ao protocolo de prevenção de lesão por pressão e queda, à lista de verificação de segurança cirúrgica e ao monitoramento indireto mensal de adesão à higienização das mãos. Em 2016, o conjunto de práticas avaliado era composto por 15 itens, 11 relativos à estrutura e quatro a processo ^5^. O penúltimo relatório divulgado, em 2023, apresentou, no inventário de avaliação, 17 itens relativos à estrutura e permaneceu com quatro referentes a processo ^6^.

Entre as práticas avaliadas, destaca-se, pela complexidade e associação com a ocorrência de falhas e EAs, o processo medicamentoso. Este responde por parcela significativa dos erros ocorrentes na assistência à saúde ^7^. A conformação dos serviços de saúde às boas práticas medicamentosas é de alta prioridade para qualidade da assistência à saúde e segurança dos pacientes.

Esse pressuposto deu origem às perguntas de pesquisa: “Estamos caminhando em direção à segurança medicamentosa?”; “Há, nos resultados de avaliação nacional, indicativos de progresso rumo à segurança medicamentosa?”. Para responder a estas perguntas, foram examinados os relatórios de avaliação de segurança do paciente, entre o início, em 2016, e o penúltimo relatório divulgado, em 2023, que totalizou oito anos de avaliação. Para tanto, foram avaliados os gráficos de Pareto de cada ano, a fim de identificar a evolução da adesão nacional às práticas de segurança medicamentosa.

Conhecer e divulgar a situação de conformidade dos serviços de saúde às práticas de segurança do paciente constitui contribuição importante para subsidiar gestores, pacientes e trabalhadores da saúde na elaboração de políticas, atitudes e processos condizentes com as necessidades de fortalecimento da cultura de segurança do paciente. Deste modo, objetivou-se identificar o grau de conformidade dos hospitais brasileiros às práticas de segurança de medicamentos, no recorte temporal de oito anos de avaliação, entre 2016 e 2023.

## Método

Realizou-se estudo descritivo, retrospectivo e documental da evolução das práticas de segurança medicamentosas no Brasil. O local de estudo abrangeu o território brasileiro, a partir do acesso aos relatórios da Avaliação Nacional das Práticas de Segurança do Paciente ^5^ ^,^ ^6^ ^,^ ^8^ ^,^ ^9^ ^,^ ^10^ ^,^ ^11^ ^,^ ^12^ ^,^ ^13^, divulgados pela Agência Nacional de Vigilância Sanitária (Anvisa) e disponíveis no sítio da Anvisa (https://www.gov.br/anvisa/pt-br). O período estudado correspondeu a oito anos de avaliação com resultados publicados de 2016 a 2023.

A principal fonte de informações foram os gráficos de Pareto nacionais, os quais registram os números de não conformidades identificadas nos serviços, relativos ao total de critérios avaliados. Esses gráficos permitem identificar as principais fragilidades, seguindo o Princípio de Pareto (80/20), para o qual, cerca de 80% dos problemas vêm de 20% das causas, chamadas de causas vitais. Cada relatório foi examinado integralmente para descobrir possíveis nuances das avaliações, possibilitando análise estratégica e direcionada às áreas que mais comprometem a segurança do paciente.

A população foi composta pelos hospitais brasileiros com leitos de UTI que responderam aos questionários de avaliação, no período de 2016 a 2023. Estima-se que o Brasil tenha cerca de 2.000 estabelecimentos de saúde com leitos de UTI ^12^. Estes vêm sendo elegíveis, desde 2016, para responderem ao questionário da Avaliação Nacional das Práticas de Segurança do Paciente.

A tabulação e apresentação gráfica dos dados foram realizadas utilizando o Microsoft Excel (https://products.office.com/). Estes foram analisados a partir de estatística descritiva simples, com uso de medidas, conforme a natureza de cada variável. As variáveis quantitativas (número de hospitais participantes, total de não conformidades e número de indicadores avaliados) foram descritas por meio de médias simples e geométrica e desvios padrão. As variáveis categóricas (como níveis de conformidade e presença de protocolos implantados) foram apresentadas em frequências absolutas e relativas (percentuais). As variações temporais (de 2016 a 2023) foram apresentadas de forma descritiva, evidenciando a tendência de evolução dos indicadores avaliados. Os cálculos e as representações gráficas foram realizados no Microsoft Excel 365.

Outrossim, a questão de interesse, a evolução das práticas de segurança medicamentosa, foi interpretada a partir da literatura pertinente, dos dados disponíveis sobre a evolução da segurança do paciente no Brasil e na América Latina e das metas nacionais e internacionais para segurança do paciente.

A pesquisa dispensou apreciação por comitê de ética em pesquisa, por utilizar dados secundários e de domínio público, sem possibilidade de identificação individual, conforme recomendado pela *Resolução nº 466/2012* do Conselho Nacional de Saúde (CNS), que trata das diretrizes e normas regulamentadoras de pesquisas envolvendo seres humanos. Ademais, encontra-se em conformidade com a *Lei nº 12.527/2011* (Lei de Acesso à Informação), que garante o acesso a dados públicos e respalda o uso de informações de domínio público sem necessidade de consentimento individual.

## Resultados

No período de 2016 a 2023, a Anvisa realizou a Avaliação Nacional das Práticas de Segurança do Paciente. Consideraram-se elegíveis para análise dos resultados os dados enviados por hospitais prioritários do país. Estes são definidos no Plano Integrado para a Gestão Sanitária da Segurança do Paciente em Serviços de Saúde, como aqueles que dispõem de leitos de UTI (adulto, pediátrica ou neonatal), conforme busca realizada no Cadastro Nacional de Estabelecimentos de Saúde (CNES) atualizado ^9^.

**Figura 1:**
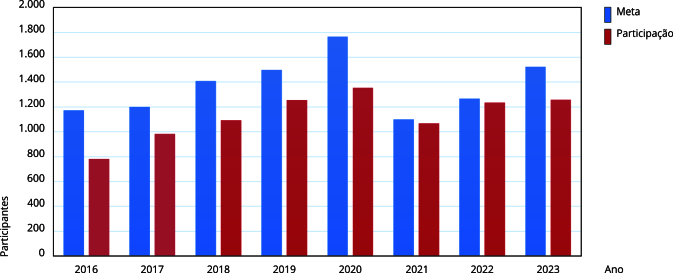
Participação dos hospitais brasileiros na Avaliação Nacional das Práticas de Segurança do Paciente no período de 2016 a 2023.

A meta para participação na avaliação foi estabelecida em 60% para o período de 2016 a 2018, e deveria atingir 90% no período de cinco anos. Esta foi recalculada no decorrer de oito anos e sofreu variações entre 60% e 90%, com média de 75 e desvio padrão de 8,87. A taxa de hospitais participantes sofreu variações entre 40% e 73%, com média de 60,8% e desvio padrão de 7,9. A Figura 1 mostra a participação dos hospitais no período examinado, com relação à meta de participação estabelecida.

Fonte: elaboração própria, a partir dos relatórios de Avaliação Nacional das Práticas de Segurança do Paciente da Agência Nacional de Vigilância Sanitária (2017-2024).

**Figura 2:**
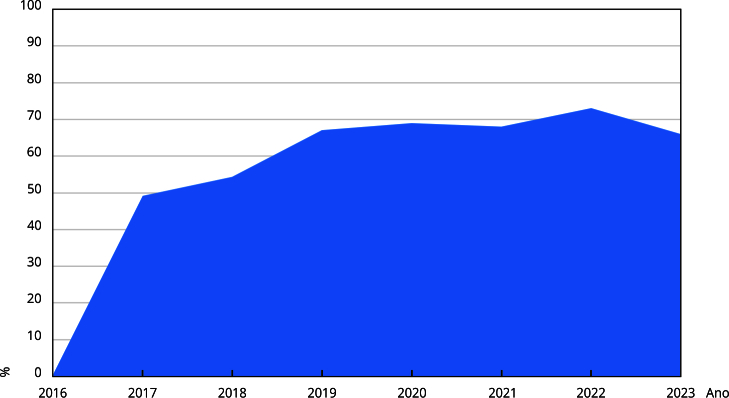
Evolução da taxa de participação dos hospitais brasileiros na Avaliação Nacional das Práticas de Segurança do Paciente.

A taxa de participação dos hospitais na pesquisa da Anvisa passou por fase de crescimento, no período entre 2016 e 2019 e a seguir, momento de estagnação até 2023, ano do penúltimo relatório divulgado (Figura 2).

Fonte: elaboração própria, a partir dos relatórios de Avaliação Nacional das Práticas de Segurança do Paciente da Agência Nacional de Vigilância Sanitária (2017-2024).

**Figura 3:**
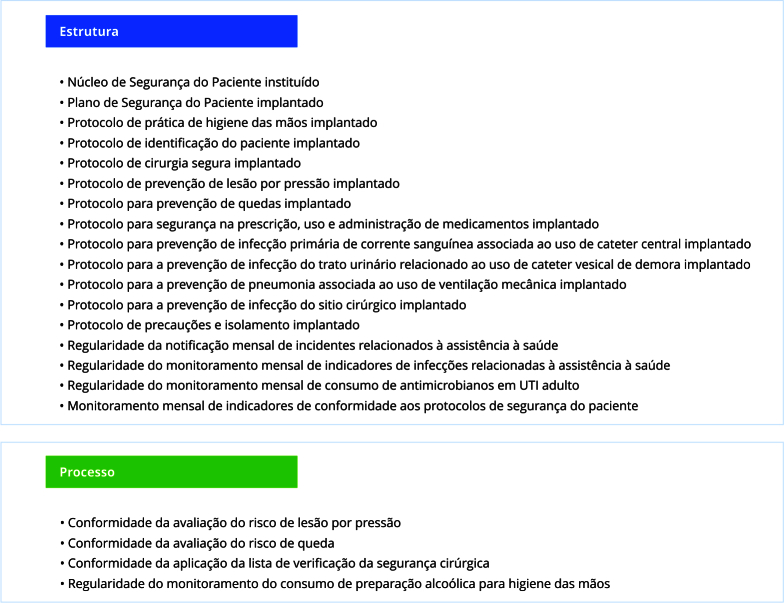
Indicadores considerados na Avaliação Nacional das Práticas de Segurança do Paciente no ano de 2023.

Quanto aos indicadores avaliados, estes inicialmente constituíram de 15, em 2016, e, ao serem revisados, atingiram, no penúltimo relatório divulgado, referente a 2023, 21 indicadores (Figura 3). Entre estes, 17 são indicadores simples, avaliam estrutura, e quatro são compostos, analisam estrutura e processo. No período examinado, a média de indicadores avaliados foi de 19,8, com desvio padrão de 1,96.

Fonte: elaboração própria, a partir dos relatórios de Avaliação Nacional das Práticas de Segurança do Paciente da Agência Nacional de Vigilância Sanitária (2017-2024).

Quanto ao processo de avaliação aplicado pela Anvisa, este foi submetido a ajustes metodológicos, ao longo do período examinado. Em 2020, foram realizadas modificações, com vistas ao aumento da confiabilidade, cuja denominação de autoavaliação foi substituída por avaliação, na perspectiva de empreendê-la, a partir do julgamento dos dados de autoavaliação, acompanhada de revisão pela Anvisa, além do sorteio de hospitais para comprovação *in loco* de amostra dos serviços participantes ^14^.

A ferramenta de análise de dados selecionada pela Anvisa para todo o período avaliado, de 2016 a 2023, foram gráficos de Pareto, os quais indicam a frequência de não conformidades em cada um dos itens avaliados. Esta ferramenta se ancora na teoria da relação 80/20, a qual sustenta que 80% dos problemas se concentram em 20% das causas, de modo que, ao se resolver 20% dessas, atenuam-se 80% dos problemas ^15^, embora a relação 80/20 trate de regra empírica, não sendo lei fixa ^16^, é fundamento que aponta o gráfico de Pareto como estratégia para identificar os chamados elementos mais vitais, cujo gerenciamento será passível de produzir grandes resultados para a organização ^17^.

**Figura 4:**
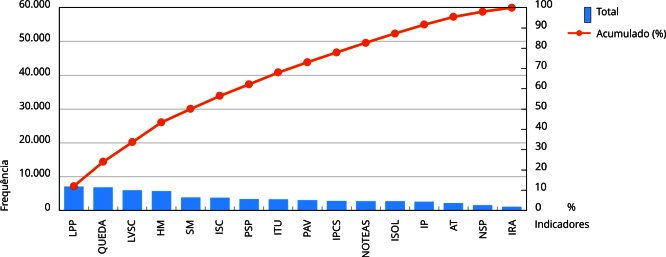
Distribuição das não conformidades do período de 2016 a 2023. Pau dos Ferros, Rio Grande do Norte, Brasil, 2024.

O total de não conformidades entre 2016 e 2023, identificadas nos gráficos de Pareto, de todos os critérios avaliados, variou de 3.233 a 13.842, com média de 7.510,7 e desvio padrão de 3.853,3. Ao considerar o método de Pareto e o total de indicadores avaliados, houve média de 12 critérios, com desvio padrão de 2,05, responsáveis por 80% das não conformidades. Essa média resulta da soma dos indicadores de todos os anos, contabilizando individualmente as variáveis dos indicadores compostos, que avaliam estrutura e processo. A Figura 4 mostra o total das não conformidades no período avaliado. Neste, os indicadores compostos foram agrupados. A Figura 4 permite identificar os 10 critérios mais vitais responsáveis por 80% das não conformidades.

AT: regularidade no monitoramento mensal de consumo de antimicrobianos em UTI-Adulto - cálculo DDD (dose diária definida); HM: protocolo de prática de higiene de mãos implantado + regularidade do monitoramento do consumo de preparação alcóolica para higiene das mãos; IP: protocolo de identificação do paciente implantado; IPCS: protocolo para a prevenção de infecção primária de corrente sanguínea associada ao uso de cateter central implantado; IRA: regularidade do monitoramento mensal de indicadores de infecções; ISC: protocolo para a prevenção de infecção do sítio cirúrgico implantado; ISOL: protocolo de precauções e isolamento implantado; ITU: protocolo para a prevenção de infecção do trato urinário relacionado ao uso de cateter vesical de demora implantado; LPP: protocolo de prevenção de lesão por pressão implantado + conformidade da avaliação do risco de lesão por pressão; LVSC: protocolo de cirurgia segura implantado + conformidade da aplicação da lista de verificação da segurança cirúrgica; NOTEAS: regularidade na notificação mensal de incidentes relacionados à assistência à saúde; NSP: Núcleo de Segurança do Paciente instituído; PAV: protocolo para a prevenção de pneumonia associada ao uso de ventilação mecânica implantado; PSP: plano de segurança do paciente implantado; QUEDA: protocolo de prevenção por quedas implantado + conformidade da avaliação do risco de queda; SM: protocolo para segurança na prescrição, uso e administração de medicamentos implantado. Fonte: elaboração própria, a partir dos relatórios de Avaliação Nacional das Práticas de Segurança do Paciente da Agência Nacional de Vigilância Sanitária (2017-2024).

Conforme a Figura 4, o indicador processo medicamentoso está entre os cinco elementos com maiores ocorrências de não conformidades. Este foi avaliado somente para o aspecto estrutura que diz respeito à existência de “protocolo para segurança na prescrição, uso e administração de medicamentos implantado”. Para comprovar esta conformidade, ao responder o relatório de avaliação, o serviço deveria anexar o documento referente ao protocolo e minimamente um comprovante de capacitação anual das equipes de saúde, em segurança de medicamentos.

**Figura 5:**
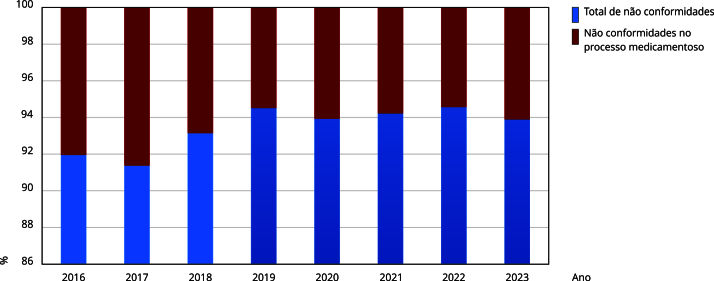
Frequência nacional de não conformidades no processo medicamentoso no período de 2016 a 2023.

No período entre 2016 e 2023, foram identificadas, aproximadamente, 3.895 não conformidades no processo medicamentoso, com média de 486,88 e desvio padrão de 219,38 para distribuição das não conformidades no período (Figura 5). Em 2017, esse foi um dos critérios que apresentou uma das menores taxas de conformidade entre os hospitais participantes, sendo responsável, isoladamente, por 9,95% das não conformidades. As regiões Nordeste, Norte e Sudeste apresentaram, até 2017, as maiores ocorrências de não conformidades no processo medicamentoso.

Fonte: elaboração própria, a partir dos relatórios de Avaliação Nacional das Práticas de Segurança do Paciente da Agência Nacional de Vigilância Sanitária (2017-2024).

Em 2018, houve queda relativa na ocorrência de não conformidades no processo medicamentoso, sustentada em cerca de 6%, até 2023. Não acompanharam esta queda três indicadores que influenciam a segurança medicamentosa, nos quais não se notou melhora significativa ao longo do tempo. Esses indicadores foram:

(a) Implantação do PSP, influente sobre todos os critérios de segurança do paciente.

(b) Regularidade da notificação de incidentes relacionados à assistência à saúde, cuja interferência sobre a segurança medicamentosa reside na constatação de que os EAs medicamentosos são rotineiros na assistência hospitalar ^18^.

(c) Adesão ao protocolo de identificação do paciente, necessário para garantir que não haja prescrição, dispensação ou administração de medicamentos para um paciente, equivocadamente.

Entre 2016 e 2019, o PSP não teve evidência entre os mais vitais, como fonte de não conformidades, à exceção de 2017. A partir de 2020, este se manifestou com taxas sustentadas entre 6% e 8% do total de não conformidades.

No que diz respeito ao indicador “regularidade da notificação de incidentes relacionados à assistência à saúde”, este foi integrado ao processo de avaliação a partir de 2020 e, desde então, mantém-se com taxas de não conformidade entre 6% e 7%. 

Já o protocolo de identificação do paciente, à semelhança do PSP, manifestou-se, persistentemente, a partir de 2020, com taxas de não conformidade entre 4% e 6%. Entre os indicadores influentes na segurança medicamentosa, a regularidade do monitoramento mensal de consumo de antimicrobianos em UTI-Adulto, incluído na avaliação a partir de 2017, mostrou-se problemática até 2019, e desde 2020, não se encontra entre os mais vitais.

## Discussão

A participação dos hospitais brasileiros com leitos de UTI na Avaliação Nacional das Práticas de Segurança do Paciente em Serviços de Saúde apresentou aumento progressivo inicial, entre 2016 e 2019, com posterior estagnação nos anos seguintes. A análise dos dados quantitativos confirma desaceleração no ritmo de crescimento da participação dos hospitais após 2019.

Entre 2016 e 2019, a taxa de participação evoluiu de 66,6% para 83,8%, com crescimento médio aproximado de 7,96% ao ano. No entanto, entre 2019 e 2023, esse ritmo não se manteve, apesar do pico pontual de mais de 97% nos anos de 2021 e 2022, período em que a meta foi reduzida. Em 2023, a taxa voltou a cair para 82,6%, revelando estagnação. A média geométrica desse período indicou leve retração de -0,4% ao ano, evidenciando a desaceleração do engajamento dos hospitais.

A redução no ritmo de crescimento de participação de hospitais brasileiros na avaliação da Anvisa, evidenciada por princípios matemáticos ou estatísticos, requer a consideração de questão complexa, na qual estão imbricadas a cultura de segurança do paciente em hospitais brasileiros e a constituição e atuação dos NQSP. A literatura aponta que a maturidade da cultura de segurança influencia diretamente o engajamento institucional e a adesão a programas de avaliação e melhoria da qualidade ^19^.

Organizações de saúde com cultura de segurança do paciente fortalecida têm mais sucesso nos sistemas de segurança, aceitam a segurança do paciente como desafio e a reconhecem como prioridade essencial, além de incorporarem a produção de relatórios à rotina ^20^. A baixa expressividade da cultura de segurança do paciente na América Latina, com percepção geral positiva de apenas 48,86% ^21^, pode suscitar conjecturas sobre a reduzida participação dos hospitais brasileiros na avaliação da Anvisa. A baixa percepção de gestores e trabalhadores sobre a importância de registrar, avaliar e relatar as práticas de segurança do paciente pode gerar compreensão equivocada do relatório da Anvisa, como apenas mais um formulário enfadonho a ser respondido ^22^.

O segundo aspecto a ser flexionado sobre essa baixa participação diz respeito à atuação dos NQSP, os quais são responsáveis pelo maior desafio da segurança do paciente, o desenvolvimento da cultura de segurança ^23^ e, entre outros, pela implementação do PSP ^24^. Esse último está entre as maiores não conformidades de 2016 a 2023, embora a implantação dos núcleos não esteja entre os maiores problemas evidenciados nas avaliações. A ausência de implantação do NQSP, representou, aproximadamente, 3% do total de não conformidades, fato que não se alinha com a taxa de 6,83% de ausência de PSP.

É importante lembrar ainda de que estes números informam somente a situação da amostra examinada e não representam a totalidade dos hospitais brasileiros. Pesquisa realizada em 2023 declara a existência de 5.000 NQSP no Brasil ^25^ para 7.400 hospitais ^26^, o que elevaria a taxa de não conformidade para implantação de NQSP, a 32,43%.

Essa situação aponta que o NQSP necessita do olhar atento dos gestores nacionais e locais para revisar constituição, valorização, condições de trabalho e grau de capacitação destes. Faltam aos núcleos disponibilidade de equipamentos básicos, espaço físico para trabalhar, recursos para implementar as ações e capacitação na área de qualidade ou de segurança do paciente ^24^ ^,^ ^27^. Muitos núcleos não têm, na composição, as categorias profissionais mínimas requeridas, enfermeiros, farmacêuticos e médicos; e não contam com profissionais com dedicação exclusiva ^28^. Essas fragilidades resultam na precarização do trabalho do NQSP e certamente comprometem o alcance das metas de segurança do paciente.

Corroboram, ainda, a necessidade de protagonizar o NQSP e a cultura de segurança do paciente, a constatação de que mais da metade dos indicadores de segurança do paciente avaliados, estão entre os mais vitais como origem de não conformidades, fato que revela a proporção 80/60 ao considerar a Teoria de Pareto e, portanto, têm-se muitas ocorrências classificadas como as mais vitais. Entre 16 indicadores ilustrados na Figura 4, dez são responsáveis por 80% das não conformidades no período avaliado. Essa situação impõe grande desafio ao gerenciamento da segurança do paciente, transformar as instituições de saúde brasileiras em serviços mais seguros.

Merecem especial atenção no foco de implementação de melhorias, os indicadores prevenção de lesão por pressão e queda, cirurgia segura, higiene das mãos, infecções relacionadas à assistência à saúde e segurança do processo medicamentoso. Este último evoluiu com redução consistente de não conformidades entre 2016 (9%) e 2019 (5,82%), mas, a partir desse ano, a melhoria parece se estabilizar em torno de 6%, com pequenas variações entre os anos, o que sugere que, provavelmente, há base sólida de melhorias, mas pode ter se atingido um platô, que exige novas medidas específicas para retomar o progresso de melhorias.

Questão que pode explicar esse possível platô é o fenômeno da pandemia por COVID-19, a qual afetou negativamente a segurança do paciente, a exemplo de declínios de melhorias de indicadores em outros países, cita-se esse declínio nos Estados Unidos ^29^. Durante dois anos, de 2020 a 2022, os sistemas de saúde direcionaram esforços e recursos para atender à alta demanda de acometidos pelo novo coronavírus que, além de impactar os hospitais com superlotação ^30^, exigiu a mobilização de tecnologias pesadas para o tratamento de pacientes com síndrome de angústia respiratória grave e outras complicações associadas à COVID-19 ^31^, o que comprometeu o investimento em prioridades concorrentes ^32^.

De outro modo, essa situação denuncia também que nossos sistemas de saúde necessitam de cultura de segurança resiliente ^29^. No Brasil, houve três condições particulares: a crise política econômica iniciada em 2015, a segunda maior carga de COVID-19 do mundo e a descoordenação do Governo Federal no período pandêmico, elementos que acarretaram enfraquecimento do Sistema Único de Saúde (SUS) ^33^ e certamente influenciaram a evolução dos indicadores de segurança do paciente no país.

No que diz respeito à segurança medicamentosa, o indicador sempre representou parcela significativa das não conformidades, com taxa média de 6%. Deve-se considerar também que a avaliação exclusivamente estrutural, sem indicador de processo, pode superestimar os resultados, tendo em vista a superioridade corrente dos aspectos estruturais sobre os de processos, situação que sugere dissonância entre protocolos formalmente implantados e processos de fato efetivados ^14^. Contudo, é legítimo enaltecer a melhoria, como também reconhecer que é necessário, ainda, muito esforço para a conquista de maior segurança no processo medicamentoso.

Para tanto, é fundamental incluir a segurança do paciente como prioridade de saúde nas políticas e nos programas do setor; buscar colaboradores na sociedade civil, universidades ou empresas, para o pleito das metas de segurança do paciente; investir no dimensionamento adequado de profissionais ^21^ e legislar a favor da proteção destes contra punições por relato de EAs ^34^; e, ainda, fortalecer ações regulatórias sobre melhores práticas, incorporar ferramentas para a segurança do paciente, além de melhorar fatores humanos e infraestrutura ^1^.

Considera-se, ainda, necessário para aumentar a segurança, designar uma pessoa ou uma equipe responsável pela segurança dos medicamentos em cada unidade ^1^ e investir em estruturas arquitetônicas orientadas para fluidez dos fluxos de trabalho, segurança e conforto de pacientes e profissionais e redução de erros e EAs ^35^.

Outros elementos reconhecidos para melhoria incluem: incorporar tecnologia da informação ao processo medicamentoso ^36^, promover treino de alta intensidade e aprendizagem em equipe sobre cultura de segurança do paciente ^20^ e garantir cargas de trabalho justas ^37^. Essas ações são reputadas pelas evidências científicas como fundamentais para melhorar a segurança do paciente globalmente e, consequentemente, o processo medicamentoso.

Apesar desses achados relevantes, este estudo apresenta duas limitações que devem ser consideradas. Primeiramente, o fato de que a maior parte dos dados são oriundos de processos de autoavaliação. Somente em 2020, a Anvisa instituiu a revisão dos dados fornecidos e a conferência das informações *in loco* em serviços sorteados. Ainda assim, volume significativo dessas informações advém de autoavaliações, o que pode influenciar na confiabilidade dos dados. A segunda limitação diz respeito à baixa participação dos hospitais na avaliação da Anvisa até 2023, fato que afeta a capacidade do estudo de fazer generalizações. Ao mesmo tempo, essa restrição permite a conjectura de que a cultura de segurança no Brasil pode ser mais frágil do que evidenciada aqui. É difícil pensar que hospitais que sequer aderem a responder um questionário online da Anvisa, tenham estruturas e processos bem fundamentados na segurança do paciente, uma vez que o alcance de metas nesse escopo e a melhoria contínua exigem constantes avaliações internas e realização de relatórios.

## Conclusão

Este estudo demonstrou que, conforme dados dos relatórios de avaliação da Anvisa, houve melhora progressiva da segurança medicamentosa em hospitais brasileiros, entre os anos de 2016 e 2019, evidenciada pela queda da ocorrência de não conformidades nesse processo, de 9%, em 2016, para 5,33%, em 2019. No entanto, a partir de 2020 - período que coincidiu com a pandemia da COVID-19 - até 2023, observou-se estagnação do percentual de não conformidades, em torno de 6%. Não obstante a melhora, o processo medicamentoso permanece como um dos itens mais críticos entre as não conformidades identificadas, o que exige intervenções direcionadas para superar esse platô.

A análise revelou, também, que, em média, 12 práticas foram consideradas mais vitais na origem das não conformidades, com grau de conformidade maior na estrutura, em comparação ao processo, o que sugere dissonância entre protocolos formalmente implantados e processos de fato efetivados. A situação abrangente dos indicadores mais vitais nas práticas de segurança do paciente no Brasil não pode ser plenamente conhecida, em face da taxa de participação real pouco superior a 50%, considerando o total de hospitais no país, com leitos de UTI.

As descobertas apontadas neste estudo sugerem a necessidade de construir serviços de saúde resilientes e adotar estratégias para retomar o avanço inicial dos indicadores de segurança do paciente, especialmente, o processo medicamentoso. Para avançar, é fundamental o comprometimento de gestores, trabalhadores e pacientes, a priorização da segurança do paciente nas políticas de saúde e o fortalecimento das ações regulatórias. O envolvimento coordenado de todos esses atores é crucial para fortalecer a cultura de segurança do paciente e consolidar práticas de saúde mais seguras em hospitais brasileiros.

## Data Availability

Os dados de pesquisa estão disponíveis mediante solicitação à autora de correspondência.
